# BML-275, an AMPK inhibitor, induces DNA damage, G2/M arrest and apoptosis in human pancreatic cancer cells

**DOI:** 10.3892/ijo.2012.1672

**Published:** 2012-10-17

**Authors:** HONG-QUAN DUONG, JAE SEOK HWANG, HEE JEONG KIM, YEON-SUN SEONG, INSOO BAE

**Affiliations:** 1 Departments of Oncology and; 2 Radiation Medicine, Lombardi Comprehensive Cancer Center, Georgetown University, Washington, DC, USA;; 3 WCU (World Class University) Research Center of Nanobiomedical Science, Dankook University, Cheonan;; 4 Department of Internal Medicine, Keimyung University College of Medicine, Daegu, Republic of Korea

**Keywords:** adenosine monophosphate-activated protein kinase, BML-275, DNA damage, G2/M arrest, apoptosis, pancreatic cancer

## Abstract

Adenosine monophosphate-activated protein kinase (AMPK) is a principal intracellular energy sensor which regulates energy producing pathways and energy requiring pathways when the cellular AMP/ATP ratio is altered. BML-275 (compound C), a well-known inhibitor of AMPK, has been found to induce apoptosis in myeloma, glioma and prostate cancer cells. However, the mechanisms responsible for the selective apoptotic effect(s) by BML-275 in cancer cells remain unknown. In the present study, BML-275 was investigated for its antitumor effect(s) in human pancreatic cancer cell lines. BML-275 inhibited the cell proliferation of 4 human pancreatic cancer cell lines (MIA PaCa-2, Panc-1, Colo-357 and AsPC-1). In addition, BML-275 significantly increased the generation of intracellular reactive oxygen species (ROS), followed by induction of DNA damage signaling and apoptosis. Furthermore, BML-275 induced cell cycle arrest in the G2/M phase. The inhibition of ROS generation by N-acetyl cysteine (NAC) significantly prevented the induction of DNA damage and apoptosis, but failed to prevent the induction of G2/M arrest by BML-275. Small interfering RNA (siRNA)-mediated knockdown of AMPKα increased the generation of intracellular ROS, DNA damage signaling and apoptosis without cell cycle arrest at the G2/M phase. These findings suggest that BML-275 exerts its antitumor effects by inducing ROS generation, DNA damage and apoptosis via inhibition of the AMPK pathway and by inducing G2/M arrest via a pathway independent of AMPK, implicating its potential application as an antitumor agent for pancreatic cancer.

## Introduction

Pancreatic adenocarcinoma is one of the most lethal and poorly understood human malignancies. Because of the lack of effective systemic therapies the 5-year survival rate for patients with pancreatic adenocarcinoma has remained at 1–3% without a change over the past 25 years (1,2). To date, the only potential curative means is surgical resection, of which only 20% of patients are eligible. Alternative therapies, such as radiotherapy and chemotherapy remain largely ineffective. Therefore, the development and evaluation of novel targeted therapeutic agents that reduce the intrinsic drug resistance of this disease poses one of the greatest challenges in pancreatic cancer research and other intractable cancers.

AMP-activated protein kinase (AMPK), a serine/threonine kinase, is a highly conserved sensor of cellular energy status in eukaryotes and is widely known as a regulator of cell metabolism (3). AMPK is a heterotrimeric protein consisting of a catalytic α-subunit and regulatory β-/γ-subunits (4,5). It is phosphorylated at Thr172 in response to an increase in the ratio of AMP-to-ATP within its activation domain of α-subunit by upstream kinases LKB1 (6–8) and calmodulin-dependent protein kinase kinase β (CaMKKβ) (9–11). Several previous studies show that excessive AMPK activation by treatment of AMPK activator (such as Metformin, 5-aminoimidazole-4-carboxamide riboside (AICAR) or A769662) inhibits the growth and/or survival of various cancer cell lines (12–19). Moreover, BML-275 (compound C), a potent, selective, and reversible ATP-competitive inhibitor of AMPK induces cell death in various types of cancers including myeloma, glioma, prostate and breast carcinoma cells (20–23). In addition, inhibition of AMPK pathway by compound C sensitizes apoptosis by co-treatment with tumor necrosis factor-related apoptosis-inducing ligand (TRAIL), doxorubicin or cisplatin in human renal, leukemia, gastric carcinoma, colon carcinoma, and cervix adenocarcinoma cell lines (24–26). Therefore, pharmacological inhibition of AMPK activity might be potentially useful in therapy of human solid tumors. However, the effect of AMPK inhibition of pancreatic cancer cell proliferation or survival has not been investigated.

Cell cycle deregulation resulting in uncontrolled cell proliferation is the one of the most frequent alterations that occurs during tumor development (27) and targeting of cell cycle progression and/or machinery is effective strategy to control aberrant proliferation of cancer cell (28,29). There are two major checkpoints, G1/S and G2/M checkpoints, are known to regulate the cell cycle. The G2/M checkpoint plays a key role in the maintenance of chromosomal integrity by allowing cells to repair DNA damage before entering mitosis. A key regulator of the cell cycle at G2/M checkpoint is cyclin dependent kinase 1 (CDK1), especially cell division cycle 2 (Cdc2). Cdc2 activation depends on the dephosphorylation of Tyr15 by Cdc25C (30). In addition, Cdc2 can be further regulated by GADD45 and 14-3-3 by p53 pathway (31). Reactive oxygen species (ROS) generation causes oxidative stress and has been shown to significantly function to controlling cancer cell survival (32). Oxidation of DNA bases and breakage of DNA strand may occurs as results of oxidative DNA damage and parts of these lesions are converted to DNA double-strand breaks (33–35). BML-275 was reported to induce cell cycle arrest at G2/M-phase and ROS generation in U251 glioma cells (22). Therefore, understanding the molecular mechanisms of BML-275 to sensitize these cells to undergo BML-275-mediated G2/M arrest and apoptosis is an important issue for effective cancer therapy.

In this study, we performed experiments to determine anti-tumor effect(s) by BML-275 in human pancreatic cancer cell lines. Our results suggest that BML-275 regulates cell survival via targeting AMPK and generating ROS in multiple human pancreatic cancer cells.

## Materials and methods

### Cell culture and reagents

MIA PaCa-2, Panc-1, CFPAC-1 and BxPC-3 cells were purchased from American Type Culture Collection (ATCC, Manassas, VA, USA) and AsPC-1, Capan-1 and Colo-357 cells were obtained from Tissue Culture Shared Resource of Georgetown University Lombardi Comprehensive Cancer Center (Washington, DC, USA). Immortal human pancreatic ductal epithelial cells, HPDE6-C7 were acquired from Dr M.S. Tsao (36). AsPC-1, BxPC-3, Capan-1 and Colo-357 cells were cultured in RPMI-1640 media supplemented with fetal bovine serum (FBS; 20% for AsPC-1, 10% for Colo-357, Capan-1 and BxPC-3 cells), 100 U/ml penicillin, 100 *μ*g/ml streptomycin and 1% sodium pyruvate. MIA PaCa-2 cells were cultured in Dulbecco’s modified Eagle’s medium (DMEM) containing 10% FBS, 2.5% horse serum (HS), 100 U/ml penicillin and 100 *μ*g/ml streptomycin. Panc-1 and CFPAC-1 cells were cultured in DMEM containing 10% FBS, 10 U/ml penicillin and 10 *μ*g/ml streptomycin. HPDE6-C7 cells were cultured in keratinocyte serum-free (KSF) medium supplemented by an epidermal growth factor and bovine pituitary extract and 1X antibiotic-antimycotic. Cell culture reagents were purchased from BioWhittaker (Walkersville, MD, USA) and Invitrogen (Carlsbad, CA, USA). BML-275 was purchased from Tocris Bioscience (Ellisville, MO, USA), and A769662 was obtained from LC Laboratories (Woburn, MA, USA).

### 3-(4,5-dimethylthiazol-2-yl)-2,5-diphenyltetrazolium bromide (MTT) assay

A total of 2,000 human pancreatic cancer cells, counted by the Luna Cell Counter (Logos Biosystems, Gyeonggi-Do, Korea) were plated in 96-well flat-bottom plates and then exposed to test the effects of BML-275 in various concentrations. At the indicated times, 10 *μ*l of 1 mg/ml MTT (Sigma, St. Louis, MO, USA) in PBS was added to each well for 4 h. After centrifugation and removal of the medium, 150 *μ*l of DMSO (Sigma) was added to each well to dissolve the formazan crystals. The absorbance was measured at 560 nm using an ELx808 Absorbance Microplate Reader (BioTek Instruments Inc., Winooski, VT, USA). Absorbance of untreated cells was designated as 100% and cell survival was expressed as a percentage of this value. Triplicate wells were assayed for each condition and standard deviation (SD) was determined.

### Western blot (WB) analysis

Cells were grown to ∼70% confluence and reagents were added at the indicated concentrations. After exposure to BML-275 alone or in combination with NAC, cells were lysed in cell lysis buffer containing 20 mM Tris-HCl, 0.5 M NaCl, 0.25% Triton X-100, 1 mM EDTA, 1 mM EGTA, 10 mM β-glycophosphate, 10 mM NaF, 300 *μ*M Na_3_VO_4_, 1 mM benzamidine, 2 *μ*M PMSF and 1 mM DTT. Protein concentrations were determined by a BCA protein assay kit (Thermo Scientific, Rockford, IL, USA). Proteins were separated by SDS-PAGE, transferred on to PVDF membranes, blocked in 1X blocking buffer (Sigma) and probed with the following primary antibodies: phospho-ACC (S79), ACC, phospho-AMPKα (T172), AMPK, phospho-ATM (S1981), phospho-CHK2 (T68), phospho-Histone H2A.X (S139), XIAP and Survivin (Cell Signaling Technology, Boston, MA), Bcl2 and Poly-ADP-Ribose-Polymerase (PARP; BD Biosciences, Franklin, NJ, USA) and α-tubulin (Sigma). Then, the membranes were incubated with horseradish peroxidase (HRP)-conjugated secondary antibodies (Sigma) and visualized with a chemiluminescence kit (Santa Cruz Biotechnology, Santa Cruz, CA, USA) according to the manufacturer’s recommended protocol and exposed with X-ray film (American X-ray and Medical Supply, Jackson, CA, USA).

### Clonogenic assay

Human pancreatic cancer cells (4x10^5^ cells) were seeded in 60-mm dishes. Twenty-four hours after plating, varying concentrations of the drugs, either as a single agent or in combination, were added to the dishes. After treatment, cells (2,000 cells) were re-seeded in 60-mm dishes (triplicate). Each culture dish was incubated for 14 days and photographed after staining with 0.5% crystal violet in 1X PBS including 25% methanol. Colonies were examined under a light microscope and counted after capturing images by scanner. Colony numbers were calculated according to the percentage of the untreated cells (37).

### Flow cytometry

Human pancreatic cancer cell lines were collected after treatment of BML-275 by trypsinization, washed with PBS and fixed overnight in 70% ethanol at −20°C. Cells were incubated with 20 *μ*g/ml propidium iodide and 40 *μ*g/ml RNase A in 1X PBS. Cells were analyzed on a FACSCalibur flow cytometer (Becton Dickinson, San Jose, CA, USA) at the Flow Cytometry and Cell Sorting Shared Resource, Georgetown University Lombardi Comprehensive Cancer Center. The acquired data were analyzed by Cell Quest Pro Analysis software (Becton Dickinson).

### Small interfering RNA (siRNA)

For the RNA interfering experiment, AMPKα-siRNA, 5′-CUGAGUUGCAUAUACUGUA-3′ and control-siRNA, 5′-GACGAGCGGCACGUGCACA-3′ were purchased from Bioneer (Daejeon, Korea). AMPKα-siRNA or control-siRNA were transfected into MIA PaCa-2 and Panc-1 cells using Lipofectamine 2000 (Invitrogen) according to the manufacturer’s procedure. After 48 h transfected cells were processed for cell cycle analysis, WB analysis and measurement of ROS generation.

### ROS generation

For measurement of ROS generation, human pancreatic cancer cell lines were treated with BML-275 with or without N-acetyl cysteine (NAC) for the indicated times and then loaded with 50 *μ*M 2′, 7′-dichlorofluorescin diacetate (DCFDA; Molecular Probes, Eugene, OR, USA) and 0.5 *μ*g/ml Hoechst 33342 (HO; Sigma) for 30 min. After rinsing, fluorescent images were taken with fluorescence intensities were obtained with a Fluorocount at excitation/emission wavelengths of 490/530 nm (DCFDA) and 340/425 (HO), and values of ROS generation were obtained by determining the ratio of DCFDA/HO signals per well.

### Statistical methods

Statistical comparisons were made using the two-tailed Student’s t-test where appropriate. Results were considered significant in at means ^*^P<0.05, ^**^P<0.01 and ^***^P<0.005. Data were expressed as the mean ± SD.

## Results

### Human pancreatic cancer cells and immortal human pancreatic duct epithelial cells express AMPKα

**Figure 1:**
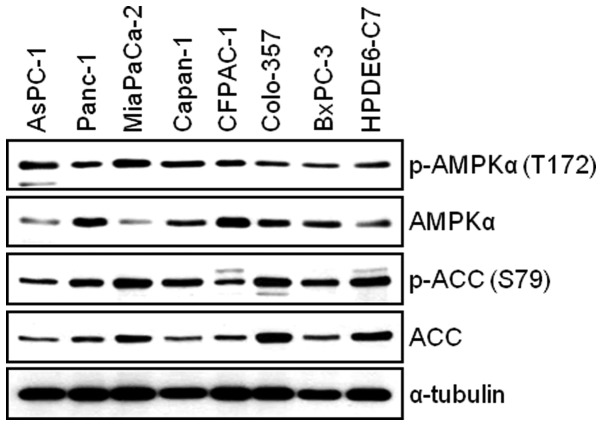
Measurements of AMPK activity in human pancreatic cancer cell lines (AsPC-1, Panc-1, MIA-PaCa2, Capan-1, CFPAC-1, Colo-357 and BxPC-3) and immortal human pancreatic duct epithelial cell line (HPDE6-C7). To avoid bias by conditions of cell growth, rapid growing pancreatic cancer cells were used for WB analysis. The total and phosphorylated forms of AMPKα (T172) and phosphorylated forms of ACC (S79) were determined. The specific phosphorylation site(s) of each kinase is indicated in parentheses. Anti-α-tubulin antibody was used for a loading and transfer control.

We first examined the total and phosphorylated form of AMPKα in AsPC-1, Panc-1, MIA PaCa-2, Capan-1, CFPAC-1, Colo-357, BxPC-3 and HPDE6-C7 cells. The WB result reveals that all pancreatic cell lines used for this study expressed the levels of both phosphorylated-AMPK and total-AMPK (Fig. 1). Next, we investigated the expression level of AMPK target protein, Acetyl-CoA Carboxylase (ACC). We found that there is relatively good correlation between the levels of phosphorylated-AMPK and phosphorylated-ACC among pancreatic cell lines (Fig. 1). To study the antitumor effect(s) by BML-275, AMPK inhibitor, in pancreatic cancer cells, we chose four pancreatic cancer cell lines: MIA PaCa-2, Panc-1, Colo-357 and AsPC-1 for further studies.

### BML-275 induces apoptotic cell death

**Figure 2:**
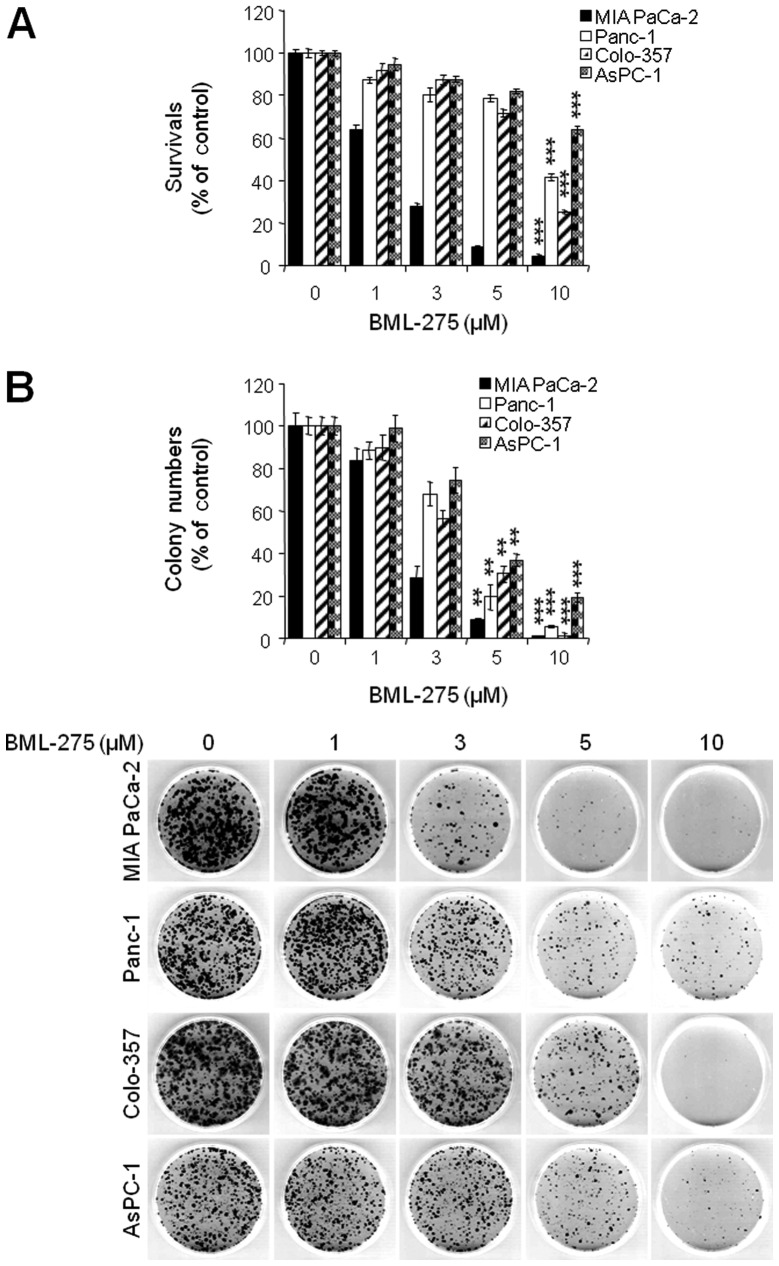
BML-275 inhibits cell viability in human pancreatic cancer cells. (A) An MTT assay of MIA PaCa-2, CFPAC-1, Panc-1 and AsPC-1 cells treated with various concentrations of BML-275 (0, 1, 3, 5 and/or 10 *μ*M) for 48 h were used to determine cell viability. Error bars represent the standard deviation. ^***^Represents statistically significant difference with p-value <0.005 between 10 *μ*M BML-275 treated group and control group. (B) A clonogenic assay of MIA PaCa-2, CFPAC-1, Panc-1 and AsPC-1 cells treated with BML-275 (0, 1, 3, 5 and/or 10 *μ*M) for 24 h was used to determine the long-term response. Colony numbers were counted and calculated as a relative percentage (%) of the untreated control cells (upper) and representative photograph of clonogenic assay results are shown (lower). Experiments were repeated 3 times and similar results were obtained. Error bars represent the standard deviation. ^**^Represents statistically significant difference with p-value <0.01 between 5 *μ*M BML-275 treated group and control group and ^***^ represents statistically significant difference with p-value <0.005 between 10 *μ*M BML-275 treated group and control group.

BML-275 is a potent ATP-mimetic competitive inhibitor of AMPK. In order to explore the antitumor effect(s) by BML-275, MIA PaCa-2, Panc-1, Colon-357 and AsPC-1 cells were treated with different concentrations of BML-275 (0, 1, 3, 5 and/or 10 *μ*M) for 48 h and cell viability were measured by MTT assay. BML-275 inhibited cell survival in dose-dependent manner (Fig. 2A). Next, we performed clonogenic assay to determine the long-term growth inhibitory effect of BML-275. Cells were treated with various concentrations of BML-275 (0, 1, 3, 5 and/or 10 *μ*M) for 1 day and continuously cultured in fresh media for 14 days and colony formation was measured by clonogenic assay. BML-275 significantly inhibited colony formation in dose-dependent manner and at 10 *μ*M BML-275 all of the tested pancreatic cancer cells showed susceptibility to the AMPK inhibitor (Fig. 2B). MIA PaCa-2 cells showed increased sensitivity to BML-275 and Panc-1 cells showed relatively less sensitive to BML-275 among four pancreatic cancer cell lines tested (Fig. 2A and B).

**Figure 3:**
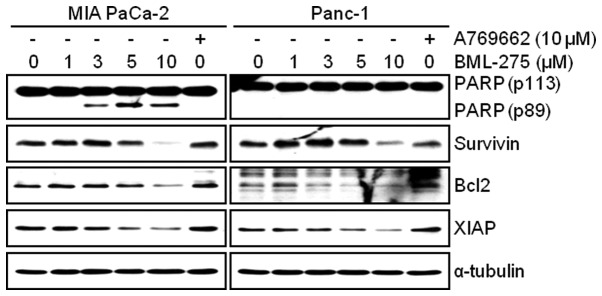
BML-275 induces apoptotic cell death. MIA PaCa-2 and Panc-1 cells were treated with BML-275 (0, 1, 3, 5 and/or 10 *μ*M) or 10 *μ*M A769662 for 24 h. WB analysis for the detection of cleaved PARP(p89) and anti-apoptotic proteins including Survivin, Bcl2 and XIAP was used to measure apoptotic cell death. The level of α-tubulin indicates a loading and transfer control.

To investigate mechanism of apoptosis by BML-275 treatment, MIA PaCa-2 and Panc-1 cells were treated with various concentrations of BML-275 or 10 *μ*M A769662 for 24 h. Apoptotic cell death was detected by WB analysis of a molecular biomarker of apoptosis, PARP cleavage. On the contrary to cells treated with A769662, cells treated with BML-275 showed an increase of cleaved PARP in MIA PaCa-2 cells but not in Panc-1 cells (Fig. 3). However, BML-275 treatment decreased the expression of anti-apoptotic proteins such as Survivin, Bcl2 and XIAP in both cell lines (Fig. 3).

### BML-275 induces G2/M arrest and sub-G1

**Figure 4:**
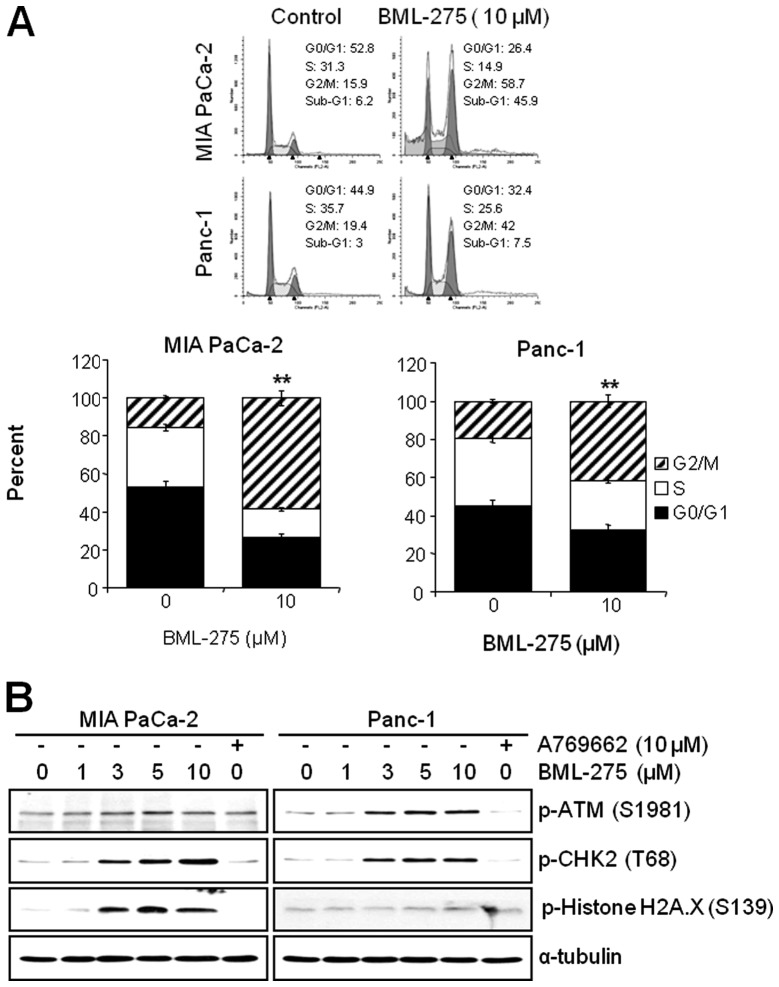
BML-275 induces G2/M arrest and Sub-G1. (A) Analysis by FACS of MIA PaCa-2 and Panc-1 cells treated with 10 *μ*M BML-275 for 24 h was used to determine cell cycle arrest. Experiments were repeated 3 times and similar results were obtained. ^**^Represents statistically significant difference with p-value <0.01 between 10 *μ*M BML-275 treated group and control group. (B) A WB of MIA PaCa-2 and Panc-1 cells treated with various concentrations of BML-275 (0, 1, 3, 5 and/or 10 *μ*M) or 10 *μ*M A769662 for 24 h was used to determine the phosphorylated forms of ATM (S1981), CHK2 (T68) and Histone H2A.X (S139). The specific phosphorylation site(s) of each kinase is indicated in parentheses. Anti-α-tubulin antibody was used for a loading and transfer control.

We next investigated if the pharmacological inhibition of AMPK by BML-275 can affect the cell cycle progression in pancreatic cancer cell lines. MIA PaCa-2 and Panc-1 cells were treated with 10 *μ*M BML-275 for 24 h and their cell cycle profiles were assessed by FACS analysis. BML-275 treatment significantly increased the cell population at G2/M-phase (from 15.9 to 58.7% in MIA PaCa-2 and from 19.4 to 42% in Panc-1) and significantly decreased the cell population at G0/G1-phase (from 52.8 to 26.4% in MIA PaCa-2 and from 44.9 to 32.4% in Panc-1) and S-phase (from 31.3 to 14.9% in MIA PaCa-2 cells and from 35.7 to 25.6% in Panc-1) (Fig. 4A). Moreover, we also observed increase of sub-G1 populations. BML-275 increased the sub-G1 population in Panc-1 (from 3 to 7.5%) and more significantly in MIA PaCa-2 (from 6.2 to 45.9%) (Fig. 4A).

DNA damage sensor CHK1/CHK2 plays a role in G2/M checkpoint via the ataxia-telangiectasia mutated (ATM)/ATM-RAD3-related (ATR) pathway. In order to further elucidate the molecular mechanism leading to BML-275-mediated G2/M arrest, we determined the activation of DNA damage signaling pathway. Treatment of MIA PaCa-2 and Panc-1 cells with BML-275 for 24 h increased the phosphorylation of ATM at Ser1981 and CHK2 at Thr68 in dose-dependent manner (Fig. 4B). These results coincide with cell cycle arrest in both cell lines. However, the phosphorylation of Histone H2A.X at Ser139, which is the molecular marker of DNA double-strand breaks, more significantly increased in MIA PaCa-2 than in Panc-1 cells (Fig. 4B). Increased levels of CHK2 and H2A.X phosphorylation were more obvious in MIA PaCa-2 cells (Fig. 4B). On the contrary, cells treated by 10 *μ*M A769662 for 24 h did not induce the phosphorylation levels of ATM, CHK2 or Histone H2A.X in either cell line.

### BML-275 decreases AMPKα activity in human pancreatic cancer cells

**Figure 5:**
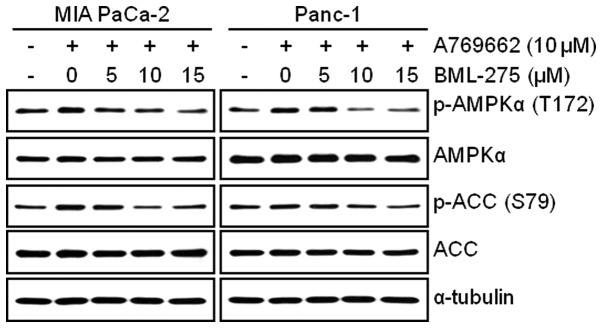
BML-275 inhibits AMPK activity. WB of MIA PaCa-2 and Panc-1 cells pretreated with 10 *μ*M A769662 for 6 h and further treated with BML-275 in different concentrations (0, 5, 10 and/or 15 *μ*M) for 24 h were used to determine the total and phosphorylated forms of AMPKα and ACC. Anti-α-tubulin antibody was used for a loading and transfer control.

In order to determine the decrease in cell survival and increase in apoptotic cell death closely correlates with the level of inhibition of AMPK activity, cells were pretreated with 10 *μ*M A769662 for 6 h and administered with various concentrations of BML-275 for 24 h. The treatment of 10 *μ*M A769662 for 6 h without BML-275 significantly activated accumulation of phosphorylated levels of AMPKα and ACC in both cell lines (Fig. 5). However, BML-275 treatment reduced the phosphorylation of AMPKα and ACC exerted by A769662 in a dose-dependent manner (Fig. 5), suggesting that antitumor effect(s) by BML-275 closely correlates with the level of inhibition of AMPK activity in human pancreatic cancer cell lines.

### The generation of ROS by BML-275 is critically required for the induction of cell death but not G2/M arrest

**Figure 6:**
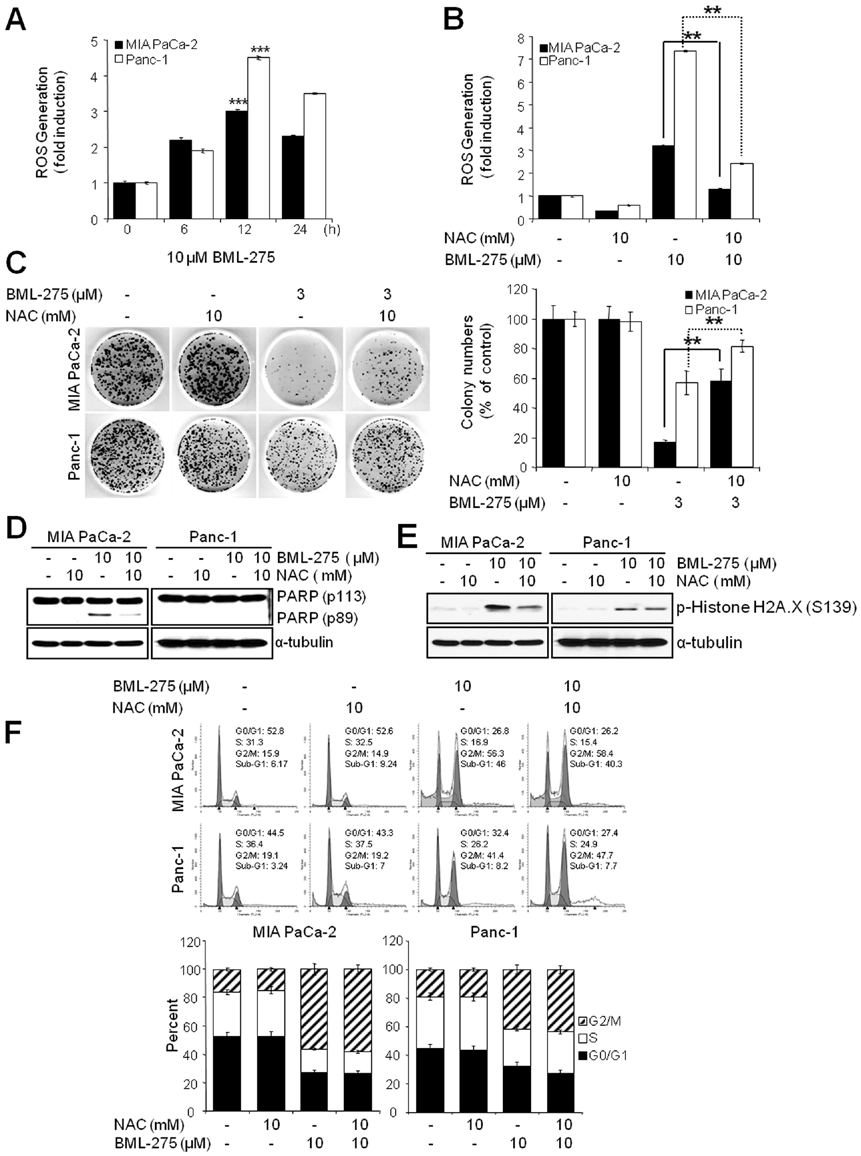
ROS generation is critically required for apoptotic cell death by BML-275. (A) The result of ROS generation analysis of MIA PaCa-2 and Panc-1 cells treated with 10 *μ*M BML-275 in different time intervals (0, 6, 12 and/or 24 h) and treated with DCFDA for a further 0.5 h to measure ROS generation. Experiments were repeated 3 times and similar results were obtained. Error bars represent the standard deviation. ^***^Represents statistically significant difference with p-value <0.005 between 10 *μ*M BML-275 12 h treated group and control group. (B) The result of ROS generation analysis of MIA PaCa-2 and Panc-1 cells pretreated with 10 mM NAC for 1 h and further treated with 10 *μ*M BML-275 for 12 h and then treated with DCFDA for further 0.5 h to measure ROS generation. Experiments were repeated 3 times and similar results were obtained. Error bars represent the standard deviation. ^**^Represents statistically significant difference with p-value <0.01 between 10 *μ*M BML-275 treated group and 10 mM NAC plus 10 *μ*M BML-275 group. (C) A clonogenic assay of MIA PaCa-2 and Panc-1 cells pretreated with 10 mM NAC for 1 h and further treated with 3 *μ*M BML-275 for 24 h was used to determine the long-term response. Colony numbers were counted and calculated as a relative percentage (%) of the untreated control cells (left) and representative photograph of colony formation assay results are shown (right). Experiments were repeated 3 times and similar results were obtained. Error bars represent the standard deviation. ^**^Represents statistically significant difference with p-value <0.01 between 10 mM NAC plus 3 *μ*M BML-275 and 3 *μ*M BML-275 group. (D) A WB analysis of MIA PaCa-2 and Panc-1 cells pretreated with 10 mM NAC for 1 h and further treated with 10 *μ*M BML-275 for 24 h was used to determine apoptotic cell death by anti-cleaved PARP antibody. (E) A WB analysis of MIA PaCa-2 and Panc-1 cells pretreated with 10 mM NAC for 1 h and further treated with 10 *μ*M BML-275 for 24 h was used to determine the phosphorylated forms of Histone H2A.X (S139). The specific phosphorylation site(s) of each kinase is indicated in parentheses. (F) Analysis by FACS of MIA PaCa-2 and Panc-1 cells pretreated with 10 mM NAC for 1 h and treated with 10 *μ*M BML-275 for 24 h was used to determine cell cycle arrest. Experiments were repeated 3 times and similar results were obtained. Error bars represent the standard deviation.

Since oxidative stress is a potent inducer of apoptosis, we next investigated if BML-275 could cause a generation of ROS in pancreatic cancer cell lines. We determined ROS generation by measuring the fluorescence of DCF which is formed by the oxidation of DCFDA by peroxides. Our results demonstrated early ROS generation by BML-275 in both cell lines (Fig. 6A). BML-275-induced ROS generation was significantly diminished by incubation with the antioxidant agent, NAC (Fig. 6B). NAC also rescued BML-275-mediated inhibition of cell survival by MTT assay (data not shown) and clonogenic assay (Fig. 6C). It also relieved the cleavage of PARP by BML-275 treatment in MIA PaCa-2 cells (Fig. 6D). BML-275-mediated phosphorylation of H2A.X at Ser139 also inhibited by NAC pretreatment (Fig. 6E). However, NAC administration did not alleviate G2/M arrest induced by BML-275 treatment (Fig. 6F), suggesting that BML-275-mediated G2/M arrest is ROS-independent at least in pancreatic cancer cell lines used for this study.

### Knockdown of AMPKα induces ROS generation and apoptosis but not G2/M arrest

**Figure 7:**
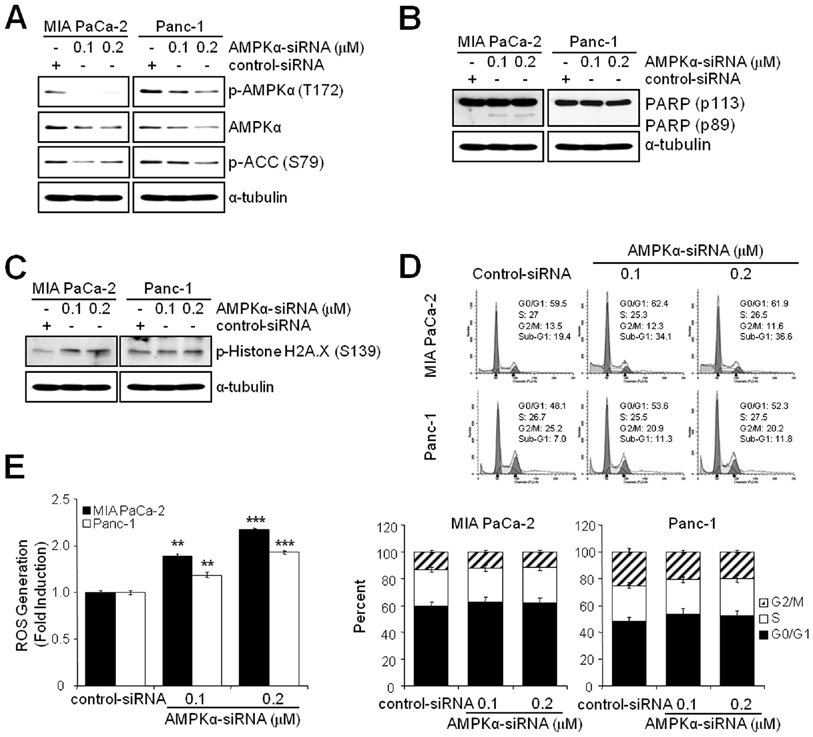
Knockdown of AMPKα induces ROS generation and apoptosis but not G2/M arrest. MIA PaCa-2 and Panc-1 cells were transfected with Control- and AMPKα-siRNA for 48 h as described in Materials and methods. (A) WB analysis was used to determine the total and phosphorylated forms of AMPKα and phosphorylated forms of ACC. (B) WB analysis for the detection of PARP cleavage was used to measure apoptotic cell death. (C) WB analysis was used to determine the phosphorylated forms of Histone H2A.X (S139). The specific phosphorylation site(s) of each kinase is indicated in parentheses. (D) Analysis by FACS was used to determine cell cycle arrest. Experiments were repeated 3 times and similar results were obtained. Error bars represent the standard deviation. (E) ROS generation analysis was used to measure ROS generation. Experiments were repeated 3 times and similar results were obtained. Error bars represent the standard deviation. ^**^Represents statistically significant difference of ROS generation with p-value <0.01 between AMPKα-siRNA (0.1 *μ*M) and control-siRNA group. ^***^Represents statistically significant difference of ROS generation with p-value <0.005 between AMPKα-siRNA (0.2 *μ*M) and control-siRNA group.

Since the inhibition of AMPK by BML-275 induced DNA damage, G2/M arrest and apoptosis in human pancreatic cancer cells, MIA PaCa-2 and Panc-1 cells were transfected with control-siRNA or AMPKα-siRNA to compare the effect(s) of BML-275 and knockdown of AMPKα. Knockdown of AMPKα with concentration of 0.1 or 0.2 *μ*M AMPKα-siRNA suppressed the level of total and phosphorylated form of AMPKα and phosphorylated form of ACC in MIA PaCa-2 and Panc-1 cells (Fig. 7A). In addition, knockdown of AMPKα also induced apoptotic cell death as evidenced by induction of PARP cleavage in MIA-PaCa-2 cells (Fig. 7B) and accumulation of sub-G1 cells in FACS analysis in MIA PaCa-2 cells (from 19.4% by control to 34.1% by 0.1 *μ*M AMPKα siRNA) but to a lesser extent in Panc-1 cells (from 7.0% by control to 11.3% by 0.1 *μ*M AMPKα-siRNA (Fig. 7D). Knockdown of AMPKα in MIA PaCa-2 cells also induced phosphorylation of H2A.X at Ser139 indicating DNA damage (Fig. 7C). The Panc-1 cells show resistance to phosphorylation of H2A.X similarly to BML-275 treatment (Fig. 4B). However, knockdown of AMPKα activity fails to display a cell cycle arrest in G2/M-phase in MIA PaCa-2 and Panc-1 cells (Fig. 7D). Finally, AMPKα knockdown induced ROS generation with increasing concentration of AMPKα-siRNA (Fig. 7E). Taken together, in pancreatic cancer cell lines, targeting of AMPKα is able to induce DNA damage, ROS generation and apoptotic cell death but not G2/M arrest.

## Discussion

In this study, we investigated the molecular mechanism of antitumor effect(s) of BML-275, an AMPK inhibitor, in human pancreatic adenocarcinoma. We found that: i) the levels of total and phosphorylated form of AMPKα and ACC vary in several different human pancreatic cancer cell lines; ii) BML-275 inhibits cell proliferation in MIA PaCa-2, Panc-1, Colo-357 and AsPC-1 cells; iii) BML-275 induces DNA damage, apoptosis and G2/M arrest; iv) the ROS generation by BML-275 is critically required for the DNA damage and apoptosis but not G2/M arrest and v) knockdown of AMPKα induces ROS generation, DNA damage and apoptosis but not G2/M arrest. This is the first report showing that BML-275 induces DNA damage, G2/M arrest and apoptosis in pancreatic cancer cell lines.

AMPK is a survival factor for cancer cells. It is involved in the augmentation of energy production through the activation of glucose uptake, glycolysis and fatty acid oxidation in response to ATP-depleting stresses (38). Solid tumors outgrowing the existing vasculature are continuously exposed to a microenvironment in which the supply of both oxygen and nutrition is limited. Previous studies showed that AMPK is critical for cancer cell adaptation in response to hypoxia or glucose deprivation (39–42). The protective role of AMPK is not restricted to nutrient stress, as this enzyme seems to play an important role in protecting tumor cells from apoptosis induced by chemotherapeutic agents such as doxorubicin, cisplatin and TRAIL (24–26). In addition, pharmacological inhibition of AMPK by BML-275 induced apoptotic cell death in myeloma, glioma, prostate cancer and breast carcinoma cells (20–23). Moreover, transfection with dominant-negative AMPK or AMPKα-siRNA was also sufficient to reduce cell proliferation of BHK, HeLa and PC12 pheochromocytoma cells or CWR22Rv1 and LNcaP prostate cancer cells (21,43). Comparing with effective apoptosis inducing dose of BML-275 treated in other cancer cell lines reported previously (20–23), most of the pancreatic cancer cell lines responded to BML-275 with different levels of responsiveness. Pancreatic cancer cell lines with relatively high level of phosphorylated AMPK showed more susceptibility to BML-275 treatment (MIA PaCa-2 and Colo-357), and those with low phosphorylated AMPK showed relatively decreased sensitivity (Panc-1 and AsPc-1).

Cancer cells usually exhibit increased levels on intracellular ROS, which in turn can initiate various cycles leading to further metabolic malfunction and ROS generation (44,45). ROS cause oxidative damage to DNA, proteins, lipids and other cellular components and therefore also significant cellular stress (45). A proposed therapeutic strategy against cancer is to treat cancer cells with pharmacological agents that have pro-oxidant properties which increase the intracellular ROS generation to a toxic threshold that triggers cell death in the cancer cells without harming normal cells (44). Vuvicevic *et al* showed that BML-275 induces ROS generation in glioma cell line, but AMPKα-siRNA treatment fails to induce ROS generation and apoptosis (22). In this study, an increased generation of ROS upon either BML-275 or AMPKα-siRNA treatment was observed and the intracellular accumulation of ROS seems to be one of critical factors in BML-275-induced apoptosis. To verify this speculation, NAC, scavenger of oxygen-free radicals, was challenged with BML-275. NAC relieved BML-275 or AMPK-siRNA mediated ROS production and improved cell viability based on the clonogenic assay, which suggested that both chemical and genetic inhibitor regulate cell viability via repressing AMPK activity.

The G2/M checkpoint plays an important role in cellular response to genotoxic stimuli. The G2/M checkpoint prevents cells from entering mitosis when DNA is damaged, providing an opportunity for repair and stopping the proliferation of damaged cells which help to maintain genomic stability (46). CHK1 and CHK2 kinases are activated at G2-phase checkpoint by DNA damage or unreplicated chromosomal DNA (47), and inactivate Cdc25C through its phosphorylation (48,49). Cdc25C was the protein phosphatase responsible for dephosphorylating and activating Cdc2, a crucial step in regulating the entry of all eukaryotic cells into the M-phase of the cell cycle. In this study, BML-275 induces cell cycle arrest at G2/M-phase possibly through the phosphorylation and activation of CHK2 kinase. The pretreatment of NAC restores the generation of ROS by BML-275 treatment in MIA PaCa-2 cell line, however, the cell cycle arrest at G2/M phase cannot be relieved, suggesting unknown effects of BML-275 or non-target effects may play a role in G2/M arrest. Previously AMPKα-siRNA treatment was reported to induce G2/M arrest in the absence of ROS generation and with no apparent cell death in U251 glioma cells (22). However, in pancreatic cancer cell line, the AMPKα-siRNA treatment induces generation of ROS and apoptotic cell death but no apparent G2/M arrest. Thus, our finding suggests that pancreatic cancer cells may be able to override the cell cycle arrest (G2/M) in response to AMPK knockdown by siRNA. On the other hand, the mechanism of DNA damage and cell death induced by BML-275 seems to be via inhibition of AMPK activity followed by stimulation of ROS production. Panc-1 is known as relatively more resistant to various antitumor agents among several pancreatic cancer cell lines (50–52). Our study also show panc-1 as more resistant to apoptotic response (cell death and PARP cleavage) upon the treatment of BML-275 and AMPKα-siRNA. Although we could not demonstrate the mechanism of resistance of Panc-1 to BML-275 treatment, this may be due to its increased multidrug resistance (MDR) gene products and/or constitutively activated cell surviving signaling pathways that confer intrinsic drug resistance (50–54).

**Figure 8:**
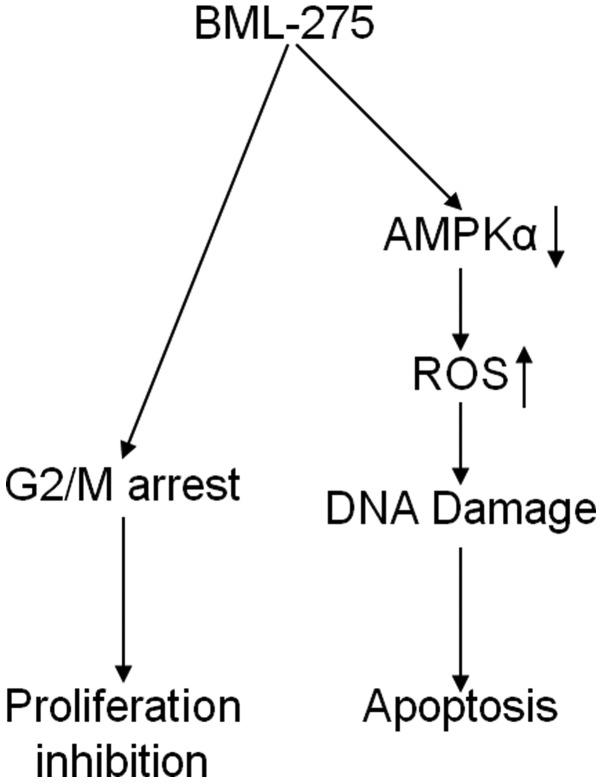
The proposed model for the mechanism by action of BML-275 in human pancreatic cancer cells. BML-275 induced DNA damage and apoptosis that is crucially regulated by ROS generation. Moreover, BML-275 caused cell cycle arrest at G2/M-phase.

In conclusion, our findings implicate that BML-275 induces DNA damage and apoptosis through AMPK-dependent mechanism and induces G2/M arrest through AMPK-independent mechanism (Fig. 8). Although the molecular mechanism of antitumor effect(s) by BML-275 requires further investigation, this compound seems to be a novel potential therapeutic agent to treat human pancreatic cancer.
